# Disentangling the roles of dopamine and noradrenaline in the exploration-exploitation tradeoff during human decision-making

**DOI:** 10.1038/s41386-022-01517-9

**Published:** 2022-12-15

**Authors:** Anna Cremer, Felix Kalbe, Jana Christina Müller, Klaus Wiedemann, Lars Schwabe

**Affiliations:** 1 Department of Cognitive Psychology, Universität Hamburg, Hamburg, Germany; 2 Department of Psychiatry and Psychotherapy, University Medical Center Hamburg- Eppendorf, Hamburg, Germany

**Keywords:** Human behaviour, Decision

## Abstract

Balancing the exploration of new options and the exploitation of known options is a fundamental challenge in decision-making, yet the mechanisms involved in this balance are not fully understood. Here, we aimed to elucidate the distinct roles of dopamine and noradrenaline in the exploration-exploitation tradeoff during human choice. To this end, we used a double-blind, placebo-controlled design in which participants received either a placebo, 400 mg of the D2/D3 receptor antagonist amisulpride, or 40 mg of the β-adrenergic receptor antagonist propranolol before they completed a virtual patch-foraging task probing exploration and exploitation. We systematically varied the rewards associated with choice options, the rate by which rewards decreased over time, and the opportunity costs it took to switch to the next option to disentangle the contributions of dopamine and noradrenaline to specific choice aspects. Our data show that amisulpride increased the sensitivity to all of these three critical choice features, whereas propranolol was associated with a reduced tendency to use value information. Our findings provide novel insights into the specific roles of dopamine and noradrenaline in the regulation of human choice behavior, suggesting a critical involvement of dopamine in directed exploration and a role of noradrenaline in more random exploration.

## Introduction

During choice, we often face the difficult decision of when to leave a known option in favor of a potentially better, but unknown alternative. While the exploitation of a known option comes with a predictable immediate reward, exploring new options is associated with a potentially higher payoff but also the risk of a low(er) reward. At the same time, exploration provides information for improving future decisions [1–3]. Extensive exploitative behavior is further linked to inflexibility and may impede gathering new information about the environment, while an extensive exploration may lead to inefficient and inconsistent decision-making, thus reducing long-term payoffs [4, 5]. Consequently, a successful adaption to complex and volatile environments requires an intricate balance of exploration and exploitation. Biases in the exploration-exploitation tradeoff have been associated with psychiatric disorders, such as addiction [6], gambling disorder [7], or anxiety disorder [8]. Given the fundamental relevance of the exploration-exploitation trade-off for adaptive behavior, understanding the mechanisms through which humans and other animals balance exploration and exploitation during decision-making is crucial.

Neural data suggest that exploration and exploitation rely on distinct brain systems, with exploitation being associated with a mechanism in the ventromedial prefrontal cortex (vmPFC) [9, 10] while exploration is linked to a track from the frontopolar cortex to the lateral PFC [2, 11, 12]. Importantly, there is accumulating evidence that exploration and exploitation not only rely on distinct neural circuits but that these processes might also be characterized by a differential involvement of major neurotransmitters, namely dopamine and noradrenaline. Striatal dopamine is commonly associated with signaling reward values and predicting future rewards [13–15]. In line with these findings, genes involved in striatal dopamine signaling were linked to exploitation [16]. However, there is also evidence suggesting a key role of dopamine in explorative behavior, associated with genes implicated in prefrontal dopamine function. Participants with a variation of the cathecol-O-methyltransferase (COMT) gene – associated with higher tonic levels of dopamine – made exploratory decisions in proportion to the uncertainty about whether alternative options might lead to better outcomes than the status quo [16]. One potential mechanism that may underlie this so-called ‘directed’ exploration is a novelty bonus that is added to unknown alternatives and may promote the acquisition of new information [17]. In line with the idea that dopamine plays a role in directed exploration, novel stimuli excite dopaminergic neurons and activate brain regions receiving dopaminergic input [18, 19].

Noradrenaline has also been repeatedly associated with exploratory behavior. For instance, high levels of noradrenaline have been shown to increase the probability of strategy shifts, whereas low levels of noradrenaline facilitate perseverative behavior [20]. In sharp contrast to dopamine, however, noradrenaline appears not to induce a bias towards information seeking when facing uncertainty (i.e., directed exploration), but rather to promote so-called ‘random exploration’ in which the induction of stochasticity leads to a value-independent exploration. Specifically, rodent studies showed that boosting noradrenaline leads to more value-free-random-like random behavior [21], whereas a pharmacological blockade of noradrenaline in monkeys resulted in increased choice consistency [22]. Noradrenaline might exert these effects by acting as a ‘reset button’ that interrupts ongoing information processing [20], thereby inhibiting the use of previously accumulated knowledge in favor of exploring new options [23].

Understanding the exact roles of dopamine and noradrenaline in the exploration-exploitation tradeoff may aid the development of new tools enabling the modulation of this tradeoff. However, to date, the distinct roles of dopamine and noradrenaline in the exploration-exploitation balance are not fully understood. Thus, the present experiment aimed to elucidate the specific roles of dopamine and noradrenaline in the exploration-exploitation tradeoff in human choice. We disentangled the involvement of dopamine and noradrenaline in specific sub processes underlying exploration and exploitation in a virtual patch-foraging task, which has been used before to dissociate exploration, operationalized as patch switching, and exploitation processes [24, 25]. Specifically, we systematically manipulated the rewards associated with the choice options, the degree to which the reward decreased, and the time it took to get to the next option. The degree to which these variables affect participants’ choice behavior may indicate to which extent explorative behavior is directed or more random.

## Materials and methods

### Participants and experimental design

Sixty-nine healthy volunteers (33 women, 36 men) between 18 and 35 years of age (mean = 24.98, sd = 3.67) were pseudorandomly assigned to one of three groups, controlling for a comparable gender allocation across groups: placebo (n = 22, 10 women), amisulpride (*n* = 23, 11 women) or propranolol (*n* = 24, 12 women). This sample size was based on a previous study examining the effect of amisulpride and propranolol on cognitive processing [26]. A-priori power analysis using G*Power [27] indicated that a sample of 63 participants is required in order to detect an effect a medium to large effect – as reported in [26]– with a power of 0.95. Because we expected a drop-out rate of up to 10 percent, we aimed at a sample size of 69 participants. Individuals with a current medical condition, current medication intake, lifetime history of any neurological or psychiatric disorder, drug or tobacco use, or intake of hormonal contraceptives in women (in order to avoid interactions with the administered drugs) were excluded from participation. Participants were further asked to refrain from caffeinated beverages and not to do any exercise on the day of the experiment. In addition, they should not eat or drink anything except water 2 h before the appointment. All testing took place in the afternoon and early evening, with the time of testing being counterbalanced across groups. All participants provided written informed consent before the beginning of the appointment and received a moderate monetary compensation. The study protocol was approved by the ethics committee of the Medical Chamber of Hamburg (PV7044).

### Pharmacological treatment

To determine the role of noradrenaline and dopamine in the exploration-exploitation tradeoff during human choice, we used a placebo-controlled, double-blind, between-subject design in which participants received orally either a placebo, 40 mg of the β-adrenoceptor antagonist propranolol, or 400 mg of the dopaminergic D2/D3 receptor antagonist amisulpride. The dosages of the drugs were based on previous studies on the role of noradrenaline and dopamine, respectively, in cognitive processes [28–31]. Because of the distinct pharmacokinetics of propranolol and amisulpride, and in line with previous studies [23, 26, 32], we administered these drugs at two separate time points. Amisulpride was administered 120 min, and propranolol 90 min before task onset. All participants received a pill at both time points, with the amisulpride group obtaining amisulpride at the first time point, followed by a placebo at the second time point and the propranolol group receiving first a placebo and subsequently propranolol. The placebo group received a placebo at both time points. Pills were indistinguishable both for the participants and the experimenter (double-blind). Participants’ intake of the pills was monitored by an experimenter.

To verify the action of the drugs, we measured blood pressure and heart rate at several time points before and after drug administration (at baseline and 90, 120, 150 and 180 min after intake of the first pill, see Fig. 2) using a digital device (OMRON model M500 (HEM-7321-D); Healthcare Europe BV, Hoofddorp, The Netherlands) with a cuff applied around the right upper arm, when participants were sitting. We took two measures (~45 s), with a 30 s interval in between. We took the raw data provided by the device and used the mean of the two measurements per time point for the manipulation check. Moreover, we measured pupil diameter and blink rate using a RED-m eyetracker (SensoMotoric Instruments GmbH) at baseline (T_1_) and 90 min after the first pill was administered (T_2_). At both time points, participants were asked to fixate a black cross, presented centrally on a gray background, for 60 s. At the beginning of the measurements, each participant’s point-of-gaze was calibrated using a 5-point calibration sequence provided by the SMI software. The software automatically returned the number of blinks counted within the 60 s and the mean pupil diameter (in mm) within this period. We did not further process the data. Changes in blink rate were quantified by the number of blinks during fixation time at T_2_ minus T_1_, and changes in pupil size were assessed by the pupil diameter at T_2_ minus T_1._

### Foraging task

**Fig. 1:**
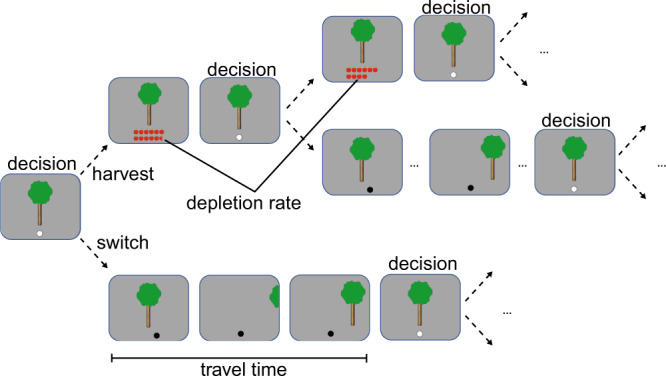
Experimental task. On each trial, participants choose whether to stay at the current tree and harvest or to switch to the next tree. Each subsequent harvest of the same tree resulted in a slightly decreased outcome and switching comes with the cost of travel time. Initial richness of trees and depletion rate differed between trees, but were equally distributed in environments with long and short travel times, respectively.

Participants performed a sequential patch-foraging task that had been used previously to dissociate explorative and exploitative behavior [24, 25]. Participants visited virtual orchards where they had to harvest apple trees with the goal to collect as many apples as possible within a limited amount of time. On each trial, they had to decide whether to stay at the current tree and harvest, or to move to the next tree (see Fig. 1). Patch switching was taken as an indicator of exploration. Each subsequent harvest of the same tree resulted in a slightly decreased return, so that at some point it was advantageous to move to the next tree. In addition to the expected reward, we manipulated the time required to reach the next tree (travel time) which was assumed to play a key role in the decision whether to continue harvesting the current tree or moving to the next tree. Travel time could be either 6 s (short) or 12 s (long) and was stable within an orchard. Participants performed four blocks, each for a fixed time of 7 min, resulting in a total task duration of 28 min. Blocks with short and long travel time orchards were alternating. Whether participants started with the short or the long travel time orchard was counterbalanced across participants and groups. The difference in travel time was used as a switching cost with switching being less advantageous in long travel times, because no apples could be collected during this time.

On each trial, participants submitted their choice via button press, using the down arrow for harvesting the currently displayed tree and the right arrow for moving on to the next tree. A white dot appeared under the tree indicating that a decision should be placed. If the participant decided to harvest the tree, the number of harvested apples was displayed after a harvest time of three seconds, followed by the white dot asking for the next decision. If the participant chose to switch to the next tree, the dot turned black and the way to the next tree was displayed, either for 6 s or for 12 s, depending on the environment.

Decisions had to be placed within 1 s, otherwise a warning appeared, followed by a short timeout before the next decision could be submitted. With each repeated harvest of the same tree, the yield of the tree decreased by a depletion rate. Each tree’s richness, i.e., the number of apples obtained from the first harvest, was randomly drawn from a Gaussian distribution with a mean of 10 and SD of 1. The depletion rate for each successive harvest of a tree was randomly drawn from a Beta distribution with parameters 14.9 and 2.0. Participants were informed that trees would vary in terms of their richness and depletion rate (i.e., some trees would be richer or poorer than others and some trees would deplete slower or faster than others), but that the trees varied in the same way across all orchards. Participants were instructed that the only factor that might change across orchards would be the time it took to travel between trees. After each block, participants could take a short break, and determine the start of the next block themselves by button press. The different blocks were distinguished by different background colors which were counterbalanced across blocks and environment types. The total number of apples harvested throughout the task was turned into payment at the end of the experiment.

### Statistical analyses

To test whether the drug manipulation was successful, blood pressure and heart rate measurements as well as eye-tracking data were analyzed using mixed-effect ANOVAs with the between-subjects factor group and the within-subject factor time. Post-hoc t-tests were used to follow-up on group differences in these measures. A mixed-effects logistic regression analysis was used to explain choice behavior. Choice was coded as stay vs. switch, indicated by 0 and 1, respectively. It was explained as a function of previous return (number of apples obtained from the previous harvest), travel time (short = 0 vs. long = 1), depletion rate, number of previous stays at current tree, and group (placebo vs. amisulpride vs. propranolol) with the placebo group as reference. We used the Akaike Information Criterion (AIC) [33] for model selection, and likelihood-ratio tests to compare our full model to gradually reduced versions. We started with a model that solely included the factor previous return and then incrementally added the factors travel time, depletion rate, number of previous stays, and group. The final model contained these five predictors, and their interaction with the experimental group (except for the factor group itself). All models consisted of the factor(s) as fixed effect(s), the overall intercept, and a random intercept per subject.

In a next step, we tested whether the factors’ estimates changed over time and whether this was different in the experimental groups. Therefore, we fitted our model separately for the first half of the task (blocks 1 and 2) and the second half (blocks 3 and 4). Note that a blockwise comparison cannot be applied here, since the blocks had either an environment with short or long travel time and these blocks were alternating. Whether the first block contained a short or long travel time orchard was counterbalanced so that an analysis based on continuous blocks would compare choices at short travel times to behavior at long travel times.

To further quantify task performance, we tested whether the total sum of rewards obtained throughout the task and the proportion of switch choices differed between the experimental groups in ANOVAs with the between factor group. In a next step, we tested whether the task performance measures differed in environments with short versus long travel times in mixed-effect ANOVAs with the between-subjects factor group and the within-subject factor travel time. All analyses were performed in R [34]. Greenhouse-Geisser correction was applied when sphericity was violated. Logistic regressions were conducted as mixed-effects models and were performed using the *lme4* package [35].

### Marginal value theorem

In an exploratory analysis, we applied the marginal value theorem (MVT) which describes the optimal behavior in patch-foraging decisions. Although the purpose of our study was not to assess whether participants used an optimal strategy, but to examine group differences in the use of information given by the task, the MVT may provide additional insights into participants behavior. Originally stated in animal literature, it assumes that an individual should leave the current option when the return falls below the average return in the environment [36]. Therefore, the optimal strategy is to switch when the expected number of apples to be obtained at the next harvest falls below the average return in the current environment:

1 $${\Bbb E}\left[ {r_{i + 1}} \right] \, < \,\rho h$$

The immediate expected reward ${\Bbb E}\left[ r \right]$ in the upcoming trial *i* + 1 results from reward in the current trial *r*, discounted by the depletion rate *κ*. The average return in the environment is reflected by the overall richness of the environment per timestep, i.e., the average reward in the current environment *ρ* multiplied by the harvest time *h*. Consequently, the MVT states that the maximum reward is yielded when participants switch at:

2 $$\kappa r \, < \,\rho h$$

Therefore, *ρh* is the threshold at which the participant should leave the current tree in favor for a new option. We simulated the optimal theshold for our task by modeling the task structure and entering all possible leaving thresholds, then probabilistically returning the expected reward over time for each threshold. We used the *optimize* function from the *stats* package in R [34] to find the exit threshold that leads to the maximum number of rewards, separately for environments with short and long travel times. For the short travel time environment this threshold is 6.7, for the long travel time environment it is 5.67. We then determined each participant’s individual leaving threshold by averaging the number of apples harvested in the last two trials before leaving to the next tree. We excluded cases in which a tree was only harvested once [25]. We used t-tests to check whether the exit thresholds in the experimental groups significantly deviated from the optimal thresholds. Further, we tested whether the exit thresholds for each environment differed between groups in an ANOVA with the between factor group.

### Computational modeling

We fitted an MVT model to our data using an error driven learning algorithm for the difference *κr*–*ρh* [24]. The model contains a learning rate *α*, an inverse temperature parameter *β*, and an intercept *c*. The average reward rate in the current environment *ρ* was updated trial-by-trial according to the difference between the actual and the expected reward *δ*, and weighted by a learning rate *α*. Note that the prediction error *δ* refers to the reward per timestep, therefore includes the time *τ* passing in the corresponding trial (harvest time *h* for stay choices, travel time *d* for switch choices):

3 $$\delta = \frac{{r_i}}{{\tau _i}} - \rho _i$$

*ρ* is updated by:

4 $$\rho _{i + 1} = \rho _i + [1 - \left( {1 - \alpha } \right)^{\tau _i}] \cdot \delta _i$$

resulting in:

5 $$\rho _i = \left( {1 - \alpha } \right)^{\tau _i}\frac{{r_i}}{{\tau _i}} + [1 - \left( {1 - \alpha } \right)^{\tau _i}]\rho _{i - 1}$$

The probability *P* for the action *a*_*i*_ was derived by the choice rule:

6 $$P(a_i = harvest) = 1/\{ 1 + \exp \left[ { - c - \beta \left( {\kappa _kr_i - \rho _ih} \right)} \right]\}$$

The learning rate *α* indicates the degree to which a prediction error leads to an adjustment of action values. It is constrained from 0 to 1 with higher values indicating a higher influence of *δ*. The inverse temperature parameter *β*, ranging from 0 to ∞ , reflects the extent to which the action values influence choice. Higher *β* values stand for more value dependent choice behavior, i.e., participants choose the option with the highest expected value, while low *β* parameters indicate value indepentent choices, i.e., random behavior. The intercept *c* can reach values from 0 to ∞ and captures any constant choice biases with higher values indicating a bias towards staying and lower values representing a bias towards switching. Please see [24] for model proof and further details. Each participant’s best fitting parameters were estimated by maximum likelihood estimation using the *optim* function in the *stats* package [34].

## Results

### Manipulation check

**Fig. 2:**
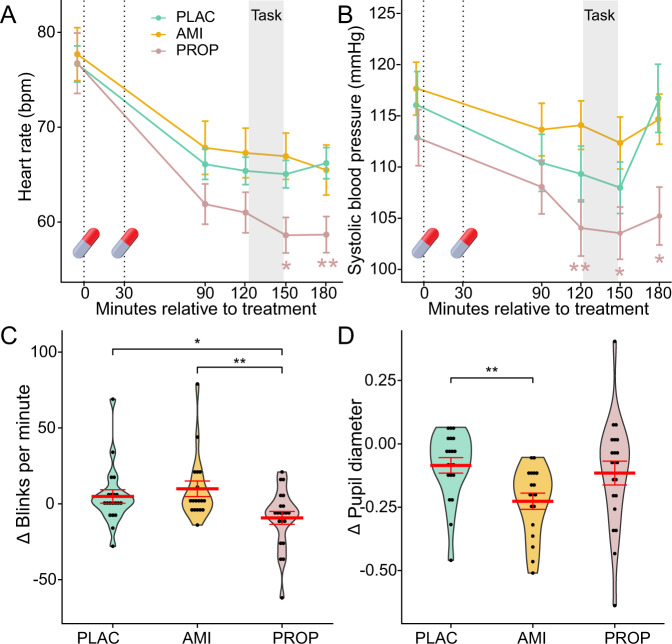
Manipulation check. The action of the drugs was confirmed by physiological measures. Heart rate and blood pressure decreased in all participants across the experiment, however, significantly more pronounced in the propranolol group, compared to the placebo group and the amisulpride group (**A**, **B**). The blink rate decreased after propranolol intake, but not after both placebo and amisulpride (**C**). A decrease of the pupil diameter was present in all groups with the strongest effect in the amisulpride group (**D**). Eye-tracking data were assessed at baseline (T_1_) and 90 min after the first pill intake (T_2_), change values reflect T_2_-T_1_. Plots show binned individual data, the width corresponds to the bin’s frequency (**C**, **D**); error bars represent the standard errors of the mean, *p < .05, **p < .01 for the post hoc t-tests between the groups.

To confirm the action of the drugs, we assessed changes in blood pressure, heart rate, blink rate and pupil diameter. Heart rate decreased in all participants across the experiment, however, significantly more pronounced in the propranolol group than in the other two groups (treatment×time: *F*(5.05, 164.09) = 3.12, *p* = 0.010 (Greenhouse-Geisser corrected), η^2^_ges_ = 0.01, Fig. 2A). Shortly before the foraging task, heart rate tended to be lower in the propranolol group, compared to both the placebo group (t(43) = −1.68, *p* = 0.099, *d* = −0.50) and the amisulpride group (t(44) −1.86, *p* = 0.069, *d* = −0.55). Immediately after the task, heart rate was significantly lower in the propranolol group than in the placebo (t(43) = −2.70, *p* = 0.010, *d* = −0.50) and amisulpride groups (t(44) = −2.70, *p* = 0.010, *d* = −0.55).

Similarly, systolic blood pressure decreased significantly more strongly in the propranolol group than in the placebo and amisulpride groups (time×group: *F*(6.43, 208.89) = 2.91, *p* = 0.008 (Greenhouse-Geisser corrected), η^2^_ges_ = 0.1; diastolic blood pressure: time×group: *F*(7.04, 228.76) = 1.21, *p* = 0.30 (Greenhouse-Geisser corrected), η^2^_ges_ = 0.008). Systolic blood pressure was significantly lower in the propranolol group than in the amisulpride group immediately before and after the foraging task (120 min after baseline: t(44) = −2.78, *p* = 0.008, *d* = −0.82; 150 min after baseline: t(44) = −2.44, *p* = 0.019, *d* = −0.72, and 180 min after baseline: t(44) = −2.53, *p* = 0.015, *d* = −0.75; Fig. 2B). Compared to the placebo group, systolic blood pressure was also lower in the propranolol group, this difference, however, was significant only 180 min after pill intake (t(43) = −2.64, *p* = 0.011, *d* = −0.79).

Blink rate differed between groups (*F*(2, 56) = 4.73, *p* = 0.013, η^2^_ges_ = 0.14) with a significant decrease from baseline to pre-task in the propranolol group, compared to placebo (t(39 = −2.29, *p* = 0.027, *d* = −0.72) and amisulpride (t(37) = −2.89, *p* = 0.006, *d* = −0.93; Fig. 2). Likewise, the pupil dilation differed between groups, but in contrast to the cardiovascular measures and the blink rate, it changed particularly after amisulpride intake (*F*(2, 56) = 3.64, *p* = 0.033, η^2^_ges_ = 0.12). As shown in Fig. 2D, pupil dilation showed a significantly stronger decrease in response to amisulpride intake, compared to placebo (t(36) = −3.20, *p* = 0.003, *d* = −1.04), and a tendency to a more pronounced decline in contrast to the propranolol group (t(37) = −1.89, *p* = 0.067, *d* = −0.61), in line with previous evidence showing an impact of amisulpride, but not propranolol [37], on pupil dilation [38]. To test whether the peripheral drug effects confounded our results, we tested whether changes in blood pressure and eye-tracking data correlated with the modeling parameters. Changes were assessed as maximum of blood pressure (systolic/diastolic) and pulse minus baseline, respectively. Changes in blink rate and pupil diameter were quantified by measures at time point 2 minus values at time point 1. None of the tests indicated an association between drug-induced changes in physiological parameters and the proportion of switch choices (all *r* < |0.13|, all *p* > 0.30), indicating that peripheral changes alone were not significantly associated with participant’s choice behavior.

### Distinct roles of dopamine and noradrenaline in human exploration-exploitation

In order to analyze the individual tendency to explore or exploit, we performed a mixed-effects logistic regression. This allowed us to (i) identify factors that influence choice behavior and (ii) examine whether these influences differ between groups. Choice was explained as a function of previous return, traveltime, depletion rate, number of previous stays, group, and the interaction of the four main factors with group. We selected this model by incrementally adding a factor and tested whether it improved the model fit, compared to the reduced version.

Separate model comparisons using likelihood ratio tests confirmed that the full model including all four main factors and their interaction with the experimental group was most appropriate. This was further reflected by the lowest (i.e., best) AIC value (Table 1).

**Table 1:** Model comparison by the Akaike Information Criterion (AIC).

Model	Model description	N params	AIC	χ^2^	df	*p*
Model 1	Previous return	3	15550			
Model 2	Model 1 + travel time	4	15413	139.49	1	<0.001
Model 3	Model 2 + depletion rate	5	15360	55.273	1	<0.001
Model 4	Model 3 + number of previous stays	6	14928	433.96	1	<0.001
Model 5	Model 4 + group	8	14930	2.026	2	0.544
Model 6	Model 5 + interactions	16	14859	86.743	8	<0.001

The full model fitted participants’ choices best in a model comparison that considers differences in model complexity. Model performance is indicated by the Akaike Information Criterion (AIC). Lower values represent a better fit. The full model contains the factors previous return, travel time, depletion rate, number of previous stay decisions for the current tree, group, and the interaction of the first four factors with the experimental group.

**Fig. 3:**
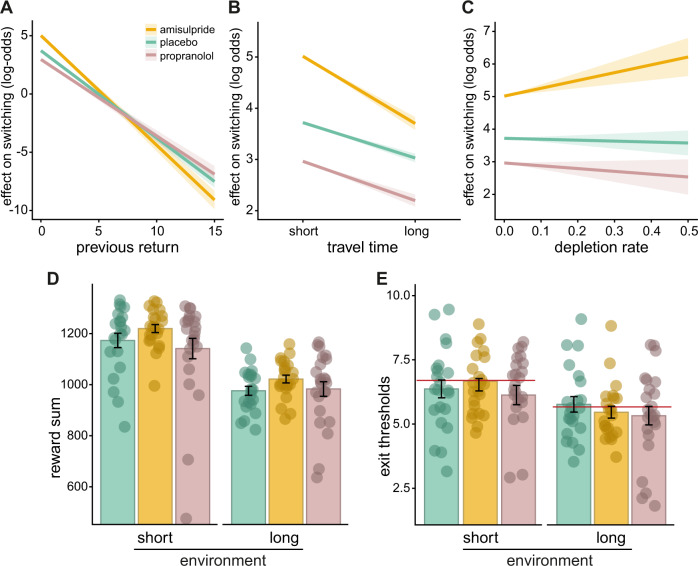
Modulation of the extent to which choice features drive behavior by amisulpride and propranolol. **A** Participants were more likely to stay at the current option when the previous reward was high and the impact of the previous reward was particularly high after amisulpride intake. **B** The impact of travel time on choice behavior was mostly pronounced in the amisulpride group, with a lower tendency to switch in particular when the travel time was long. **C** While the depletion rate did not influence choices in placebo and propranolol groups, participants in the amisulpride group switched in particular in the face of a high depletion rate (**D**). The amisulpride group tended to collect a higher number of rewards in environments with short travel times, compared to the propranolol group (t(45) = 1.80, *p* = 0.078, *d* = 0.53). In blocks with long travel times, participants tended to collect more rewards after amisulpride intake, compared to placebo (t(43) = 1.97, *p* = 0.055, *d* = 0.59). **E** Exit thresholds differed between environments, but not between groups, red lines reflect the optimal exit threshold in the short and in the long travel time environment, following the marginal value theorem (MVT). Please note that the critical difference between the amisulpride and propranolol groups (**A**–**C**) remains if the two participants with the lowest reward sum (**D**) are removed from the analysis. Error bars represent standard errors of the mean.

The mixed-effect logistic regression indicated that previous reward and travel time had significant effects on choice behavior. Participants in all three groups switched less when previous returns were high (main effect of previous reward, β = −0.749, *z* = −21.259, *p* = <0.001). Importantly, however, this effect was differently pronounced in the groups. Compared to placebo, the amisulpride group switched significantly less often when previous rewards were high (previous return×amisulpride: β = −0.192, z = −3.625, *p* < 0.001). In sharp contrast to the amisulpride group, the propranolol group switched more often after high rewards, compared to placebo (previous return×propranolol: β = 0.092, *z* = 1.956, *p* = 0.050, Fig. 3A).

Furthermore, as expected, a long travel time was associated with less switching (main effect of travel time, β = −0.691, *z* = −8.214, *p* = <0.001). This effect, however, was more pronounced in the amisulpride group, compared to placebo (travel time×amisulpride: β = −0.623, z = −4.948, *p* < 0.001), indicating that the amisulpride group was particularly reluctant to switching in the face of a long travel time. The propranolol group, in turn, did not differ from the placebo group (β = −0.076, *z* = −0.644, *p* = 0.520). The depletion rate alone did not have an impact on choices, neither in the placebo group (main effect of depletion rate: β = −0.287, *z* = −0.376, *p* = 0.701), nor in the propranolol group (depletion rate×propranolol: β = −0.574, *z* = −0.531, *p* = 0.595). Interestingly, in interaction with amisulpride a higher depletion rate was associated with a higher probability to switch (depletion rate×amisulpride: β = 2.685, *z* = 2.298, *p* = 0.022, Fig. 3C).

We further tested whether the choice behavior developed throughout the task by fitting the model separately for the first half of the task (blocks 1 and 2) and for the second half (blocks 3 and 4). In general, both the results of the first and second half are in line with the overall analysis. Participants switched less when the previous return was high (first half: β = −0.80, *z* = −15.49, *p* < 0.001; second half: β = −0.87, *z* = −15.81, *p* < 0.001), and when the travel time was long (first half: β = −0.56, *z* = −4.69, *p* < 0.001; second half: β = −0.91, *z* = −7.24, *p* < 0.001). The influence of the depletion rate, however, emerged throughout the task – in the first half it did not influence choice behavior (β = 0.31, *z* = 0.29, *p* = 0.77), while in the second half participants switched even more when the depletion rate was low (β = −3.16, *z* = −2.68, *p* = 0.007). Interestingly, this analysis points towards overall behavioral biases both in the amisulpride and in the propranolol group. In the first half, the amisulpride group showed a significantly enhanced switching behavior, compared to the placebo group (β = 2.36, *z* = 2.58, *p* = 0.010), while the propranolol group did not differ from placebo (β = −0.32, *z* = −0.38, *p* = 0.70). In the second half, however, the propranolol group switched less than the placebo group (β = −1.6, *z* = −1.88, *p* = 0.061), while the amisulpride group did not differ from placebo (β = 0.97, *z* = 1.06, *p* = 0.29). Other than that, the results confirm the findings from the overall analysis: the amisulpride group switched less, when the previous return was high (first half: β = −.0.28, *z* = −3.50, *p* = 0.0005; second half: β = −0.22, *z* = −2.67, *p* = 0.008) and when the travel time was long (first half: β = −1.17, *z* = −6.29, *p* < 0.0001; second half: β = −0.34, *z* = −1.85, *p* = 0.06). Likewise, participants in the amisulpride group switched more when the depletion rate was high (first half: β = 3.25, *z* = 1.93, *p* = 0.05; second half: β = 3.64, *z* = 2.06, *p* = 0.04, Supplementary Fig. S2 in the Supplementary Material). Again, neither of the choice factors significantly influenced decision making in the propranolol group.

### Task performance

Groups did not differ in the number of total rewards obtained throughout the task (*F*(2,66) = 1.68, *p* = 0.19, η^2^_ges_ = 0.048). However, participants in the amisulpride group tended to collect more rewards, compared to placebo (t(43) = 1.92, *p* = 0.061, *d* = 0.57) and propranolol (t(45) = 1.65, *p* = 0.11, *d* = 0.48). The number of rewards differed between environments with short and long travel time (main effect of travel time: F(1, 66) = 229.85, *p* < 0.0001, η^2^_ges_ = 0.36, Fig. 3), but there was no significant interaction between environment and experimental group (*F*(2,66) = 1.176, *p* = 0.31, η^2^_ges_ = 0.006). In environments with short travel times, the amisulpride group tended to yield higher rewards, compared to the propranolol group (t(45) = 1.80, *p* = 0.078, *d* = 0.53). In long travel time environments participants tended to earn more rewards after amisulpride intake than after placebo (t(43) = 1.97, *p* = 0.055, *d* = 0.59; all other *p* > 0.15, Fig. 3D). Overall, the groups did not differ in the percentage of switch decisions (*F*(2,66) = 0.48, *p* = 0.62, η^2^_ges_ = 0.14). The percentage differed between environments with short and long travel times (main effect of travel time: *F*(1, 66) = 36.89, *p* < 0.0001, η^2^_ges_ = 0.056), but this was not differentially pronounced in the experimental groups (group×travel time: *F*(2, 66) = 1.03, *p* = 0.36, η^2^_ges_ = 0.003; all post hoc t-tests *p* > 0.16).

### Marginal Value theorem

Exit thresholds differed between environments (main effect of travel time: *F*(1,66) = 47.70, *p* < 0.0001, η^2^_ges_ = 0.072) but did not differ between groups (*F*(2, 66) = 0.37, *p* = 0.69, η^2^_ges_ = 0.01). There was no group×travel time interaction (*F*(2,66) = 1.27, *p* = 0.29, η^2^_ges_ = 0.004). Neither group differed from the optimal exit threshold, as supposed by the MVT (6.7 for short travel times environments, all *p* > 0.76; 5.67 for long travel time environments, all *p* > 0.85, Fig. 3E).

### Computational modeling

**Fig. 4:**
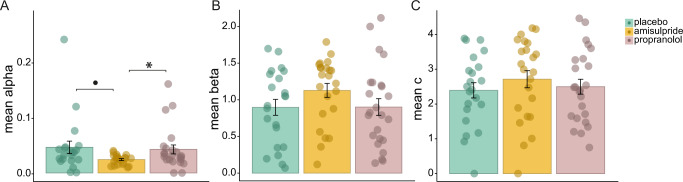
Modeling parameters per subject. The learning rate *α* was significantly lower in the amisulpride group than in the placebo group and tended to be lower, compared to the propranolol group (**A**). Neither the temperature parameter *β*, nor the choice bias *c* differed between the experimental groups (**B**, **C**), error bars represent standard errors of the mean, * for *p* < 0.05,• for *p* < 0.06.

We fitted a computational model according to the MVT to estimate each participant’s learning rate *α*, temperature parameter *β*, and choice bias *c*. Regarding the learning rate, we identified three participants as outlier, as they differed more than 3 standard deviations from the group’s mean (one participant from each experimental group). Interestingly, participants in the amisulpride group had a significantly lower learning rate than participants in the propranolol group (t(43) = −2.16, *p* = 0.036, d = −0.65, Fig. 4A), and tended to have a lower *α* compared to the placebo group (t(41) = −1.99, *p* = 0.054, *d* = −0.61, Fig. 4). The learning rates did not differ between the placebo and propranolol groups (t(42) = 0.28, *p* = 0.78, *d* = 0.083).

The temperature parameter *β* did not differ between groups (F(2,66) = 1.53, *p* = 0.22, η^2^_ges_ = 0.044, Fig. 4B). Neither the amisulpride nor the propranolol group differed significantly from the placebo group (amisulpide vs. placebo: t(43) = 1.6, *p* = 0.12, *d* = 0.48; propranolol vs. placebo: t(44) = 0.028, *p* = 0.98, *d* = 0.008; amisulpride vs. propranolol group: t(45) = 1.51, *p* = 0.14, *d* = 0.44). Likewise, the choice bias *c* did not differ between groups (F(2,66) = 0.51, *p* = 0.6, η^2^_ges_ = 0.020, Fig. 4C). Neither the amisulpride group, nor the propranolol group differed from placebo (amisulpride vs. placebo: t(43) = 0.97, *p* = 0.34, *d* = 0.29; propranolol vs. placebo: t(44) = 0.34, *p* = 0.73, *d* = 0.10; amisulpride vs. propranolol: (t(45) = 0.67, *p* = 0.51, *d* = 0.19).

## Discussion

Adaptive decision-making requires an optimal balance between choosing known options and trying new paths when the environment changes or new information is required. Given the ubiquity of exploration-exploitation tradeoffs in everyday life and their potential relevance for psychopathology, understanding the mechanisms involved in this tradeoff is important. Here we investigated the specific roles of dopamine and noradrenaline in the exploration-exploitation tradeoff by pharmacological blockade of either system using propranolol and amisulpride and systematically examining the effects of reward values, depleting returns, and opportunity costs on choice behavior. The action of the administered drugs was confirmed by specific changes in blood pressure, heart rate, pupil dilation, and blink rate. As expected, (systolic) blood pressure and heart rate decreased most prominently in the propranolol group, consistent with its action as a hypotensive agent [38], related to the blockade of β1- and β2-adrenergic receptors that represent the predominant form of adrenergic receptors expressed in the heart [39]. Propranolol was further linked to a reduced blink rate, which may be due to dryer eyes after β-adrenergic blockade [39]. Pupil diameters, in turn, known to be mediated, at least partly, by dopaminergic neurons in the ventral tegmental area (VTA; [40]) were particularly reduced in the amisulpride group, most like due to the blockade of D2/D3 receptors in the VTA [37, 41]. Most importantly, our behavioral results revealed functionally dissociable roles of dopamine and noradrenaline in the exploration-exploitation trade off, with dopamine governing the sensitivity to decision-relevant information and noradrenaline being involved in value-independent choice processes.

Previous studies suggested a role of dopamine in exploration [4, 17, 19, 42]. Our data, however, do not point towards a decrease of exploratory behavior in participants that received the D2/D3 receptor antagonist amisulpride. Instead, participants in the amisulpride group switched less, specifically when (i) the previous reward was high, (ii) the travel time was long, and (iii) the depletion rate was low. This pattern suggests an increased sensitivity to the specific choice aspects, i.e., that these had a stronger impact on choice. These results corroborate previous findings showing that D2-receptor blockade by amisulpride sharpened content-specific representations in the PFC that are used to guide reinforcement-based decisions [29, 32]. Interestingly, in the first half of the task, the amisulpride group showed significantly enhanced switching behavior, compared to the placebo group, indicating an increase in explorative choices. Taken together, these results point towards a directed exploration in the beginning of the task, which may then inform subsequent choice behavior. This is further supported by our computational modeling results. Participants in the amisulpride group had a lower learning rate compared to the other groups. Given the strong local autocorrelation of prediction errors in the present foraging task, a low learning rate may be beneficial to integrate across a longer time span. In line with our data, recent findings suggested that cabergoline, a D2 receptor agonist, reduced the sensitivity towards the difference between rich and poor environments [43]. Assuming that a D2 receptor blockade should impair dopamine-associated processes, these findings might be puzzling at first glance. However, the potential discrepancy between these findings and common beliefs about the role of dopamine in choice could be explained by a dual state model of prefrontal dopamine. This model proposes that the activation of prefrontal D1 and D2 receptors has opposing effects on GABAergic activity, resulting in bidirectional effects on the accuracy of prefrontal representations [44]. In recordings of prefrontal pyramidal neurons, a predominant D1 receptor activation (D1-dominated state) was associated with increased GABAergic inhibition, resulting in a selective access to prefrontal circuits with only very strong inputs passing through and therefore forming strong representations. A primary D2 receptor activation (D2-dominated state), on the other hand was linked to a decreased GABAergic inhibition so that multiple inputs were processed at the same time, leading to weak representations in the prefrontal cortex [44]. It is assumed that blocking prefrontal D2 receptors increases the likelihood of D1-dominated states, i.e., the processing of strong input while suppressing noise [45]. Further, amisulpride is suggested to preferably block D2/D3 receptors in the PFC, while dopamine levels in the striatum were even increased after low doses [41, 46, 47]. Our findings may thus be explained by a shift towards prefrontal D1 receptor activation in the prefrontal cortex, which may, together with an intact striatal dopamine functioning, lead to the formation of strong representations of decision-relevant stimuli and ultimately increased sensitivity for specific choice aspects to guide behavior.

In sharp contrast to the amisulpride group, none of these choice aspects had a significant effect on choice behavior in the propranolol group. Interestingly, participants in the propranolol group tended to switch even more after higher rewards, compared to the placebo group. Specifically, they still switched less after higher than lower rewards, but this was less pronounced than in the placebo group, while this effect was significantly more pronounced in the amisulpride group than in the placebo group. This pattern points to a reduced usage of decision-relevant information for choice behavior, in line with evidence suggesting a role of noradrenaline in random, but not directed exploration [21–23, 48]. However, the data on the direction of noradrenergic effects on random exploration is heterogenous. A recent study directly compared how amisulpride and propranolol affect different exploration strategies and reported that propranolol, but not amisulpride attenuated random exploration [23]. This is in line with previous findings showing that noradrenaline levels predicted increased noise in choice behavior [49]. Our data suggest an opposite effect of noradrenaline on decision noise with rather increased noise after blocking noradrenaline. The present findings dovetail with a study that reported decreased random exploration after pharmacologically elevated noradrenergic activity [48]. In the same vein, it was hypothesized that noradrenaline might work as an urgency signal that promotes commitment to an early decision. Noradrenergic blockade via propranolol was assumed to insert this signal and hence stop further information gathering [50]. This is further supported by our finding that, in the second half of the task, participants in the propranolol group showed an overall reduction of switch choices, pointing again towards a reduced use of information, but in the direction of exploitative decision-making. These heterogeneous results with respect to the direction of the influence of noradrenaline on exploration and exploitation might be related to distinct activity modes of noradrenaline. While tonic noradrenergic activity was associated with exploration, phasic noradrenaline has been thought to facilitate exploitative behavior [51]. Because there is evidence that propranolol is likely to influence both tonic and phasic signaling of noradrenaline [52], such differentiation cannot be derived from our data.

In addition to differences between tonic and phasic noradrenergic activity, a possible inhibitory mechanism of β-adrenergic receptors may explain why we found a tendency towards an increase of stochasticity. Specifically, β-adrenergic receptors enhanced inhibitory synaptic mechanisms in rats by a noradrenaline-mediated enhancement of GABA efficacy [53]. By blocking β-adrenergic receptors, we might have blocked a noradrenaline-related inhibition of noise, resulting in an increase of noisy, i.e., random behavior. This was not captured by a decreased temperature parameter in the propranolol group. However, the general range of the temperature parameter derived by the modeling approach was rather low, which can be explained by the low range in the value estimation. The temperature parameter specifies the degree to which value estimates influence behavior. Since the initial rewards were drawn from a Gaussian distribution with a mean of 10 and SD of 1, depleting by a Beta distribution with parameters 14.9 and 2.0, the estimated values came in a low range per se. Consequently, the degree to which this estimation influenced decision-making may not be suitable to interpret group differences in this case.

Overall, however, the influence of propranolol on the exploration-exploitation tradeoff was less pronounced than for amisulpride. A potential explanation for this could be that noradrenaline does not drive specific components of decision-making, but rather exerts higher-order control signals, such as an urgency signal that stops ongoing information gathering, presumably by inducing decision noise.

At this point, it should be noted that other factors such as tiredness or boredom might have affected switching behavior. Although these factors may also contribute to more random exploration and we do not think that these could explain the influence of the drugs on the dependency of switch behavior on relevant decision parameters, future studies should measure these additional variables to explicitly control for their influence. Moreover, future studies should consider including baseline measures of task performance to rule out performance differences between groups before drug administration or use a within-subject design instead of a between-subjects design.

Taken together, our findings suggest functionally dissociable roles of dopamine and noradrenaline in the exploration-exploitation tradeoff during human decision-making. Compared to placebo, participants in the amisulpride group switched less when the prospects in the current environment were still advantageous (i.e., high rewards and low depletion rates) and the costs associated with exploration were high (i.e., long travel time). After propranolol intake, participants tended to switch even more, compared to the placebo group, when the rewards in the current environment were still high. Thus, these data show that dopamine modulates the sensitivity to choice relevant aspects, while noradrenaline regulates when to disengage from the current information paths to randomly explore new options. Our results are thus generally in line with previously hypothesized roles of dopamine and noradrenaline in directed and random exploration, respectively. The present findings enhance our understanding of the differential roles of dopamine and noradrenaline in decision-making and might have relevant implications for mental disorders characterized by biases in the exploration-exploitation tradeoff.

## Supplementary information

supplemental material
