# Supplementary material for: Disentangling the roles of dopamine and noradrenaline in the exploration-exploitation tradeoff during human decision-making
Source: Neuropsychopharmacology. 2022 Dec 15;48(7):1078–86. doi: 10.1038/s41386-022-01517-9 (PMC10209107; doi:10.1038/s41386-022-01517-9)
Supplement: Supplementary file 1 — supplemental material [file 41386_2022_1517_MOESM1_ESM.pdf]

SUPPLEMENTAL MATERIAL

Disentangling the roles of dopamine and noradrenaline in the exploration-exploitation tradeoff during human decision-making

Anna Cremer[1], Felix Kalbe[1], Jana Christina Müller[2], Klaus Wiedemann[2], and Lars Schwabe[1]

[1] Department of Cognitive Psychology, Universität Hamburg, Germany

[2] Department of Psychiatry, University Clinic Hamburg- Eppendorf, Germany

Corresponding Author:

Prof. Dr. Lars Schwabe

Universität Hamburg

Department of Cognitive Psychology

Von-Melle-Park 5

20146 Hamburg, Germany

Tel.: +49 40 43838-5950

Fax.: +49 40 42838-4729

E-mail: lars.schwabe@uni-hamburg.de

Analysis of Response times

As an exploratory analysis, we tested whether response times differed between groups. Overall, response times did not differ between groups ($F(2,66)=0.28$, $p=0.76$, $\eta^2_{ges}=0.008$, all post-hoc t-tests $p>0.46$). Response times differed between short and long travel time environments (main effect of travel time: $F(1, 66)=8.75$, $p=0.004$, $\eta^2_{ges}=0.005$), but there was no significant interaction with the experimental group (group×travel time: $F(2, 66)=1.33$, $p=0.27$, $\eta^2_{ges}=0.002$; all post hoc t-tests $p>0.33$).

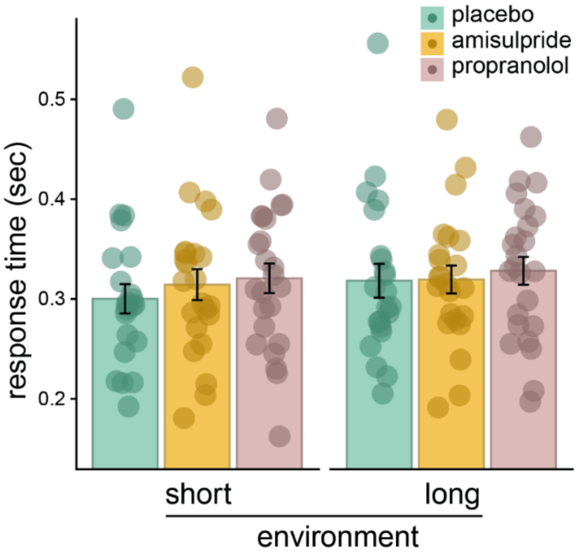

Figure S1. Response times differed between environments with short and long travel times, but there was no significant interaction with the experimental group; all p>0.33).

Analysis of choice behavior throughout the task

We tested whether choice strategies evolved during the course of the task. This analysis revealed a general choice bias of both the amisulpride and the propranolol group which was not observable in the overall analysis. In the first half, the amisulpride group showed a significantly enhanced switching behavior, compared to the placebo group ($\beta=2.36$, $z=2.58$, $p=0.010$), while the propranolol group did not differ from placebo ($\beta=-0.32$, $z=-0.38$, $p=0.70$). In the second

half, however, the propranolol group switched less than the placebo group (β=-1.6, z=-1.88, p=0.061), while the amisulpride group did not differ from placebo (β=0.97, z=1.06, p=0.29).

Neither total rewards, nor exit thresholds differed between groups in the first and second half of the experiment.

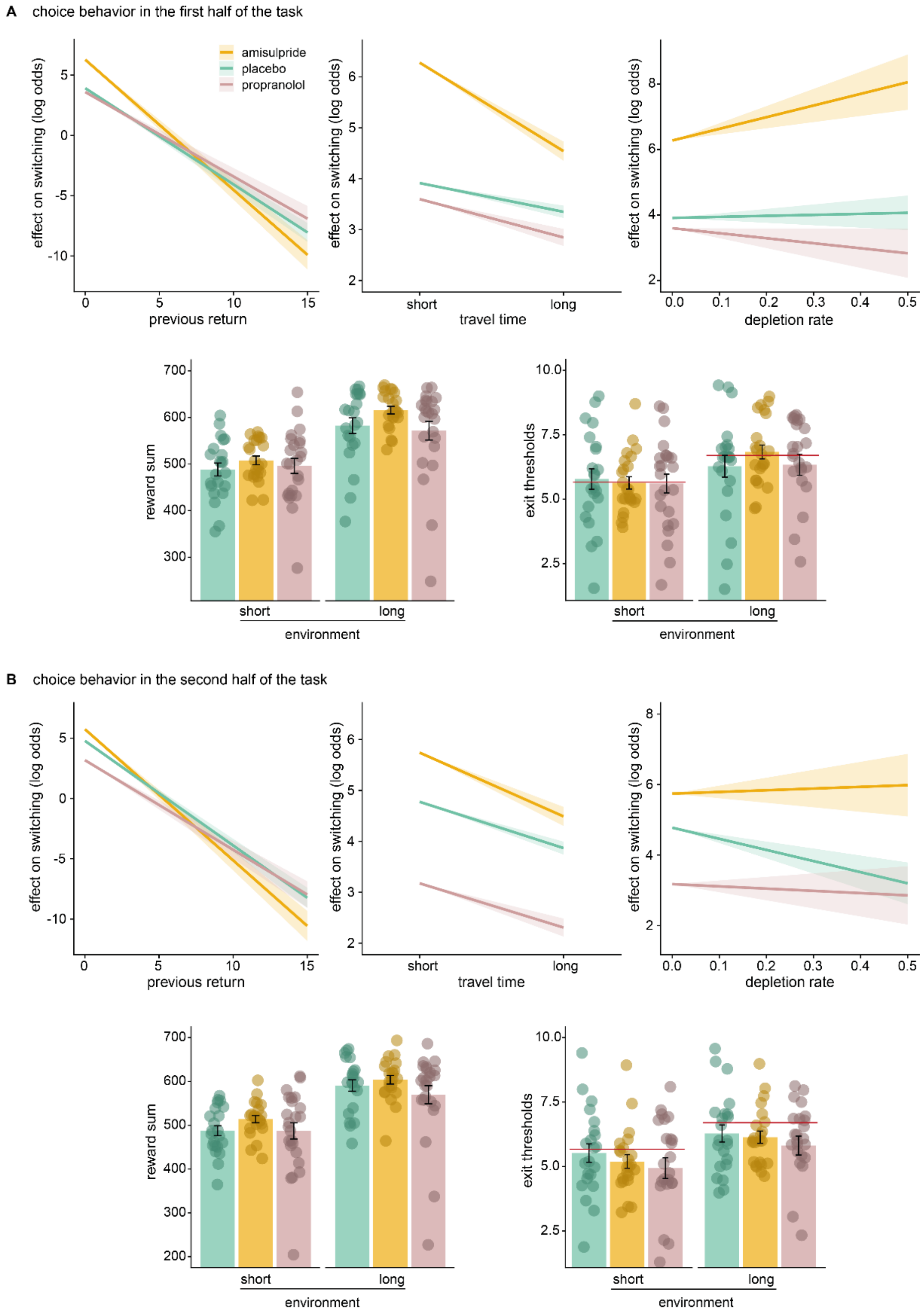

Figure S2. Development of choice behavior throughout the task. (A) In the first half, the amisulpride group switched significantly more often, compared to placebo, pointing towards an explorative choice strategy to collect information. (B) Contrary, the propranolol group differed from placebo in the second half of the task where participants in the propranolol group switched less than the placebo group indicating an exploitative choice behavior. Overall, task performance did not differ between the first and second half of the task.
